# Infrared spectroscopic analysis of restorative composite materials' surfaces and their saline extracts

**DOI:** 10.1186/2194-0517-2-9

**Published:** 2013-03-18

**Authors:** Reem Ajaj, Robert Baier, Jude Fabiano, Peter Bush

**Affiliations:** 1 Section of Biomaterials, Division of Conservative Dental Sciences, School of Dentistry, King Abdulaziz University, Jeddah, 22254 Saudi Arabia; 2 Department of Oral Diagnostic Sciences, Division of Biomaterials, State University of New York at Buffalo, 355 Squire Hall, 110 Parker Hall, Buffalo, NY 14214 USA; 3 Department of Restorative Dentistry, State University of New York at Buffalo, Buffalo, NY 14214 USA

**Keywords:** Multiple attenuated internal reflection-infrared, Resin composite, Human gingival fibroblasts, Forensic, Cytotoxic

## Abstract

This study aims at finding out if multiple attenuated internal reflection-infrared (MAIR-IR) spectroscopic analysis can be used as a tool to differentiate commercial resin composite brands and to find out if different resin composites will have different abilities of leaching materials that are cytotoxic to human gingival fibroblasts (HGFs) Tooth-colored resin fillings have become increasingly popular as restorative materials, which make it important to differentiate the commercial brands for forensic and biological purposes. Fourteen resin composite brands were used in the study. MAIR-IR spectroscopic analysis was used for surface characterization of the organic and inorganic parts of the resin composite samples which were studied as is and after 2 weeks of saline incubation. IR spectroscopy was also done on the saline extracts to find out if different resin composite materials would have different leaching abilities. The saline extracts were also used for the viability testing of HGF cell cultures. One-way analysis of variance test statistics was used to analyze the results. It was found that the resin composite brands have different spectra after saline soaking. It was also found that these resin composite brands possess different leaching abilities with regard to the amount and type of materials and different cytotoxic effects, which were found to be threshold dependent, meaning there is a critical or threshold value of leaching material at or above which the toxic effect will be significant and below which there is no toxic effect. Therefore, IR spectroscopy might be considered as a useful tool for dental resin composite characterization. However, more oral simulating environmental testing methods, different surface characterization methods, and more cell viability testing methods and assays must be considered for more specific results which relate more to the behavior of these dental resin composites in the oral environment.

**Electronic supplementary material:**

The online version of this article (doi:10.1186/2194-0517-2-9) contains supplementary material, which is available to authorized users.

## Introduction

Recent literature has demonstrated how the slightly different inorganic fractions of dental resin composites maybe used for forensic identifications of unknown accident victims, but has not examined the possible additional identifying value from examination of the resinous organic fractions of these same materials. Similarly, certain dental restorative resinous materials have been implicated in providing saline-extractable components that were toxic to human gingival fibroblast cells (HGFCs), but the identities of these extractable substances have not been revealed by analysis. Recognizing that infrared (IR) spectroscopic analysis of both the resin composites and their saline extracts could provide surface-sensitive information relevant to both the forensic and possible biotoxicity issues previously raised, this investigation set out to determine if IR spectroscopy using the multiple attenuated internal reflection (MAIR)-IR technique could serve these needs. For forensic scientists, it might add to the database collected previously using other methods that led to the use of the portable generator-based X-ray fluorescence (XRF) instrument for nondestructive analysis at crime scenes (Jeffrey et al. 2005) and the Spectral Library for Identification and Classification Explorer (Bush et al. 2008; Ubelaker et al. 2002). For clinicians, it might aid in the appropriate selection for clinical use depending on their cytotoxic behavior.

The main instrumental approach used in our study was MAIR-IR spectrometry for surface compositional analysis of 14 resin composite brands; all of them were included in previous studies of resin composites (Bush et al. 2006, 2007a, 2008; Hermanson et al. 2008). IR spectroscopic analysis was done on the resin composite samples ‘as is’ and after saline soaking for 2 weeks in an incubator under 37°C to simulate body intra-oral conditions. Saline soaking of the samples was done to evaluate possible surface compositional changes that might occur after these restorations are placed in the patients' mouths. Lee et al. (1995a) reported changes in the infrared spectra of the surfaces of these composites after immersion in 75% ethanol and in artificial saliva (Moi-Stir, Pendopharm, Montreal, Canada). Vankerckhoven et al. (1982) used MAIR-IR spectroscopy to determine the influence of some manipulative factors (polymerization time, temperature, and mechanical treatments such as polishing) on the concentration of unreacted methacrylate groups in scrapings from the surfaces of the resin composites, and all of the tested manipulations caused a decrease in the resin composites' apparent surface double-bond content. A review of the literature did not identify any prior studies that have used MAIR-IR spectroscopy to examine the intact resin composite surface chemistry of as-prepared or saline-extracted resins, as they would appear in the oral cavity.

The second aspect in our study was the IR spectroscopic analysis of the saline extracts of the resin composites. Studying the saline extracts of these composites is significant to know if different brands of resin composites have different leaching abilities with regard to the amount and type of the leached materials and thus have potentially different toxicities to cells in the proximal vicinity of resin composite restorations in the mouth. Evidence of leaching from various fillers has been reported using plasma spectrometry (Soderholm 1983) and atomic absorption spectrophotometry (Soderholm et al. 1984; Soderholm 1990). Leached components from dental composites in oral simulating fluids have also been studied using gas chromatography/mass spectrometry (Lee et al. 1998).

The third aspect of our study was cytotoxicity testing of the saline extracts of the resin composites. This was accomplished by adding the saline extracts to HGFs and using a widely accepted viability and proliferation test method, methylthiol tetrazolium (MTT) assay (Wikipedia, 2012), to obtain the results. The fact that some proportions of residual monomers or short-chain polymers may not react and remain un-bonded after curing of dental composites, in addition to the susceptibility of polymers in dental resin restorations to chemical degradation (Lee et al. 1998), makes it crucial to understand how these materials might react in the biological environment. Thompson et al. (1982) used ultraviolet spectrophotometry to analyze the un-polymerized materials extracted from cured orthodontic bonding resin in various aqueous solutions and found that orthodontic bonding resins, even when mixed and cured according to the manufacturers' instructions, do leach considerable amounts of un-polymerized components and that precautions should be observed during the polymerization and handling of these materials. High-pressure liquid chromatography was used to analyze different commercial resin composites for the presence of bisphenol-A (BPA) and/or bisphenol-A dimethacrylate (BAD) (estrogen-like components), assuming that these materials could contribute to the overall estrogen load that might result in deleterious side effects, but it was concluded that dental resins in general do not represent a significant source of BPA or BAD exposure (Lewis et al. 1999; Schmalz et al. 1999).

Components eluted from dental resin composites, including diluents (triethylene glycol dimethacrylate (TEGDMA) and decamethacrylate) and some additives (ultraviolet stabilizer TINUVINP), plasticizers (dicyclohexyl phthalate and bis(2-ethylhexyl) phthalate), initiator (triphenyl stibine), coupling agent (γ-methacryloxypropyl trimethoxysilane) and phenyl benzoate, have been shown to make collagen less resistant to trypsin digestion (Lee et al. 1998). Trypsin is an enzyme that acts to degrade protein (proteolytic enzyme or proteinase) (Infoplease, 2012). Collagen is a very important component structure of the bone, teeth, and the gingival and periodontal ligament, all of which can be affected when restorations are placed in contact with or near them. Collagen is produced by fibroblast cells (including HGF). It has also been well established that the resin composite co-monomer TEGDMA causes gene mutation in some cases *in vitro* (Schweikl et al. 2006).

## Methods

Fourteen composite samples were collected from commercial sources (Prisma AP.H, SureFil, Quixx, and Esthet.X (Dentsply Caulk, Milford, DE, USA); 4 Seasons, Tetric Evo Ceram, and Heliomolar (Ivoclar Vivadent, Amherst, NY, USA); Filtek Supreme Plus (3M ESPE, St. Paul, MN, USA); Durafill VS and Venus (Heraeus, South Bend, IN, USA); Grandio (VOCO, Cuxhaven, Germany); ICE and Rok (SDI, Bayswater, Australia); and 3D-Direct (Brea, CA, USA). Four samples from each resin composite brand were made, two for use in MAIR-IR spectroscopic analysis and the other two for saline incubation and further analysis of the samples and saline extracts using MAIR-IR spectroscopy (Perkin-Elmer (Waltham, MA, USA) Spectrum 100 FTIR spectrophotometer, with Perkin-Elmer ATR mirror assembly). The samples were made using a mold (ResinKeeper) for composites, manufactured by COSMEDENT^®^ (Manalapan, NJ, USA), and light cured for 40 s using a Spectrum^®^ 800 curing unit (DENTSPLY Caulk) operating at an intensity of approximately 550 mW/cm^2^ of halogen light. Two samples from each brand were used for the as-is spectral analysis and another two for the ‘saline immersion’ and further spectral analysis.

### Infrared spectra of the resin samples as is

The MAIR-IR spectroscopic instrument was adjusted during all procedures with the IR spectra wave number ranging from 4,000 to 600 cm^-1^, transmission in percentage, 10× scan, and 4-cm^-1^ resolution. Two samples were used for each resin composite brand and were clamped to the KRS-5 prism. After sample removal, the readings of the residues were taken (no residues were found).

The other two resin composite samples from each resin composite brand were placed in 45-ml conical tubes and immersed in 10 ml of 0.9% sodium chloride (physiologic saline) solution. They were placed in the incubator (37°C) for 2 weeks and shaken at random times. After the 2-week period, samples were removed from the saline solution using pre-cleaned tweezers and placed on labeled microscopic glass slides under a fume hood until the samples were dry.

### Infrared spectra of saline-soaked samples

The same procedures for the as-is samples were applied. Also, the spectra were subtracted from their own baselines (using the spectral subtraction option provided in the instrument software) and converted to absorbance mode then baseline corrected by choosing the ‘automatic baseline correction’ option in the software. Bands were located, and the heights and bases of the peaks were recorded for calculation of the absorbance of each peak.

### Infrared spectra of the saline extracts

For each resin composite material's extract, a standard analytical procedure was applied as follows: 500 μl of the composite saline extract was placed on the germanium prism (does not dissolve in water) using a 100-μl Eppendorf Digital Pipette 4710 (Eppendorf, Hauppauge, NY, USA) and then placed under the fume hood until drying was complete; spectrum of the saline extract was then taken as is, after distilled water leaching, and after distilled water rinsing. The protocol for distilled water leaching was to apply distilled water until it covered the surface of the prism, leaving it for 15 s, and then spilling it, followed by air drying. For distilled water rinsing, distilled water was delivered from a squeeze bottle for 15 s by holding the prism about 20 cm away to produce a shear stress of approximately 1 Pa, and again air drying.

Also, the spectra of the composite saline extracts were converted to absorbance mode, then baseline corrected. Bands were located, and the heights and bases of the peaks were recorded for calculation of the absorbance of each peak.

### IR spectroscopy of reference materials

The following materials were collected from commercial sources and are known constituents of the dental resin composite compositions:

- *Ethylene dimethacrylate (EDMA)* cross-linking monomer (Lot no. 283–11, Polyscience, Inc., Rydal, PA, USA)
- *Ethylene glycol dimethacrylate 98% (EGDMA)* (Lot no. 05216CI, Aldrich Chemical Company, Inc., Milwaukee, WI, USA)
- *95% TEGDMA* (Lot# 110 k3657, Sigma^®^, Seelze, Germany)
- *Bis-A-dimethacrylate* (Lot no. 03924AR, Aldrich Chemical Company, Inc.)
- *(1S)-(+)-Camphorquinone (d-2,3-bornanedione)* (Lot no. 58H3516, Sigma^®^, Germany)
- *(1R)-(-)-Camphorquinone 99%* (Lot no. 04129TI, Aldrich Chemical Company, Inc.)

Instrument settings were adjusted as described previously. For EDMA, EGDMA and TEGDMA, these monomers were spread over the germanium prisms, and the spectrum for each of them was taken. For the bis-A and camphorquinones, these materials were in powder form and dissolved in acetone to be placed on the germanium prisms. The infrared spectrum of acetone alone, after evaporation, showed no infrared absorption. Acetone was used to dissolve the materials and then placed on the germanium prisms, and the spectra of these materials were taken after thorough drying.

### Scanning electron microscopy/energy-dispersive spectroscopy of the saline extracts

An amount of 500 μl of each composite's saline extract was dried on a germanium prism. scanning electron microscopy/energy-dispersive spectroscopy (SEM/EDS) of one specimen (Prisma AP.H) was taken and showed the presence of no elements other than Na, Cl, and Ge. Prisma AP.H was selected randomly, as a typical sample from the larger group. SEM pictures and EDS analysis were taken for three different areas on the germanium prism randomly selected.

### Viability testing

**Figure 1:**
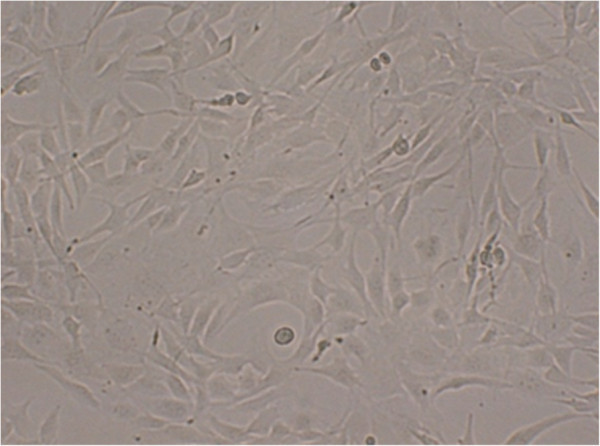
**HGF cells after growing to confluence, viewed under a light microscope.** ×40 original magnification.

Culture medium for the HGFs was prepared using 5 g of minimum essential medium (Alpha medium) from GIBCO™ (Cat. no. 12000–041, Lot no. 397128, Life Technologies, Grand Island, NY, USA), 1.1 g of sodium bicarbonate, 5 ml of L-glutamine 200 mM 100X, 5 ml of antibiotic-antimycotic penicillin-streptomycin, and 50 ml of fetal bovine serum (JM Biosciences, San Diego, CA, USA). Cured resin composite's saline extracts for each brand were filter sterilized using 5-ml syringes (BD Luer-Lok™ Tip, Franklin Lakes, NJ, USA; latex free, sterile) and a 0.45-μm polyvinylidene difluoride filter (Acrodisc LC GELMAN^®^, Pall, Port, Washington, NY, USA) that fits into the tip of the syringe. The control was pure saline, and the samples were filter sterilized directly before adding them to the cell cultures. Cell cultures were grown to confluence for 10 days (Figure 1).

**Figure 2:**
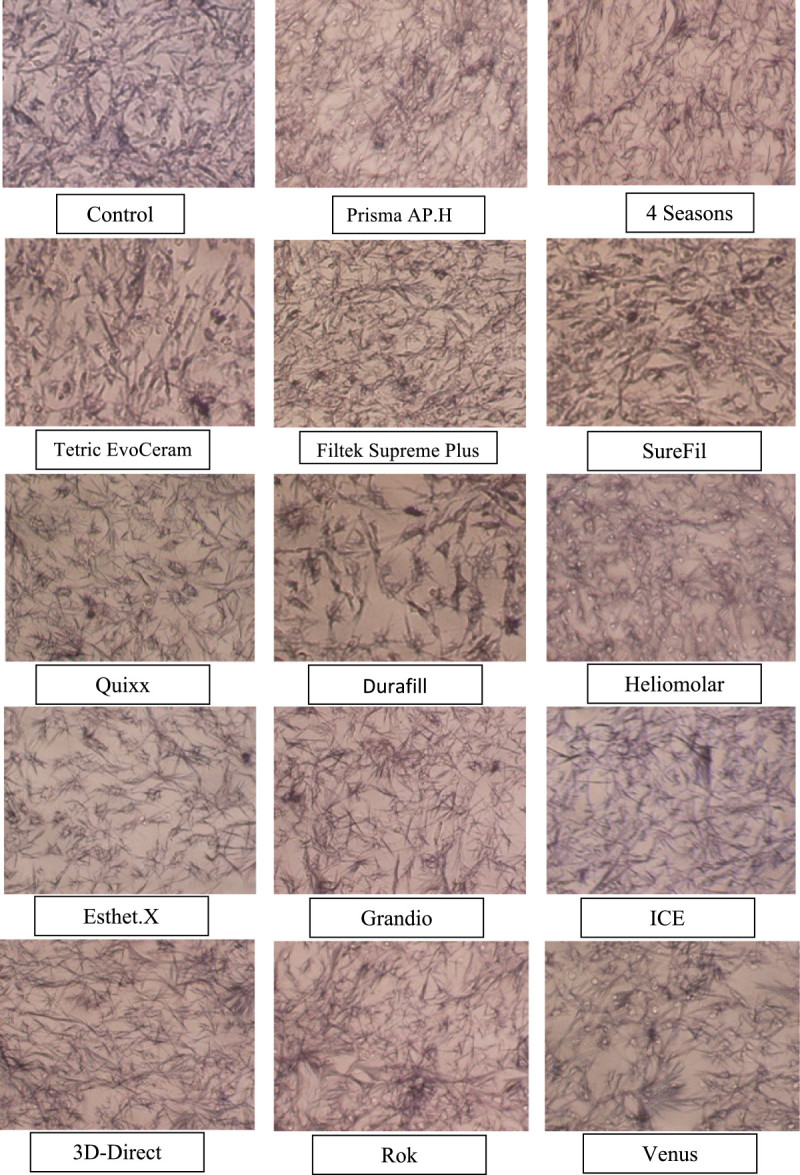
**MTT precipitate for the 50-μl added composite saline extracts group under a light microscope.** ×40 original magnification.

Cell cultures were replaced into 24-well cell culture plates; each well contained 500 μl of cell culture media. An amount of 50 μl from each extract was filter sterilized and added to the seeded cells (after removal of 50 μl of cell culture media from each well). For the control and each resin composite extract, the experiment was done in triplicate. Forty-eight hours later, 50 μl of the MTT reagent was added to each well. Twenty-four hours later, examination of the cell cultures under a light microscope showed the purple precipitate in all cultures (Figure 2). Cells were transferred to a 96-well microplate with 200 μl of cell culture in each well to enable reading of the formazan titer in the microplate reader machine. The plate was placed in the microplate reader, set at 595 nm wavelength, and readings were taken.

**Figure 3:**
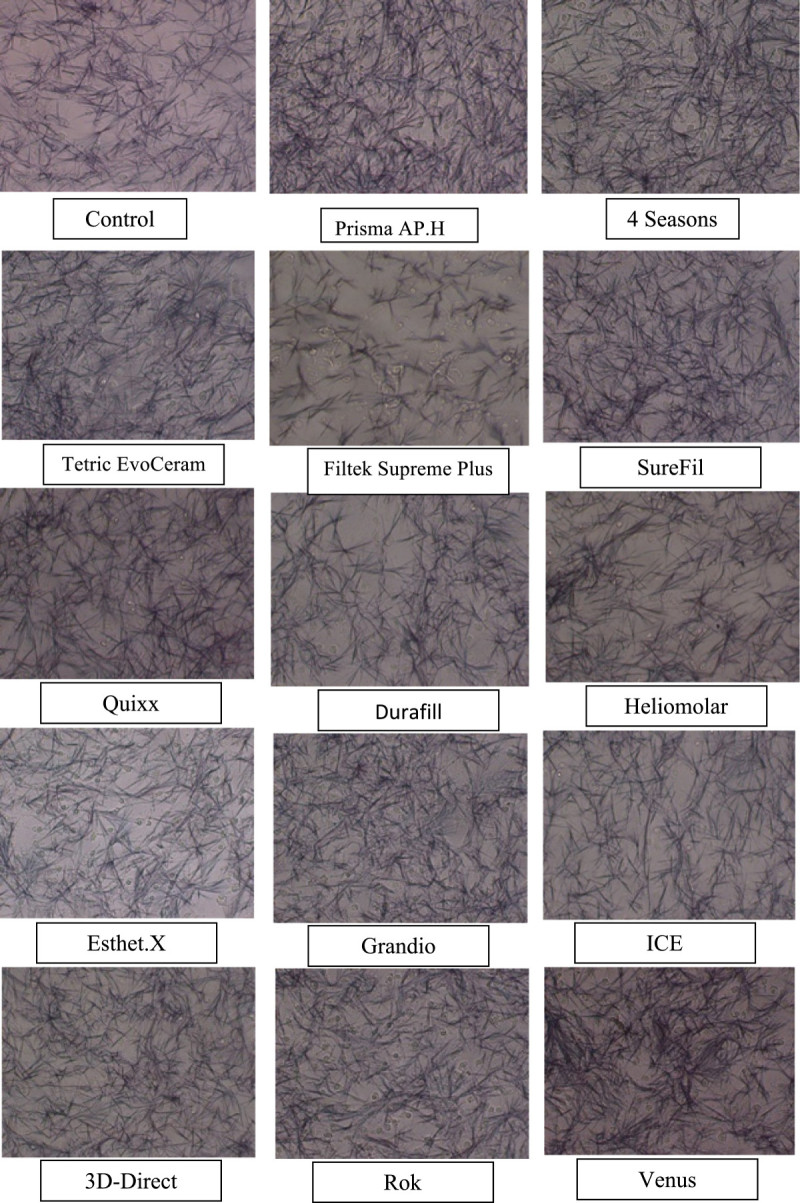
**MTT precipitate for the 100-μl added composite saline extracts group under a light microscope.** ×40 original magnification.

New cell cultures were grown as described above. All steps were repeated the same way, but 100 μl of the composite saline extracts were added to 400 μl of medium in each well. MTT assay was repeated the same way, and photos of the purple formazan precipitate under a light microscope (×40 magnification) were taken (Figure 3). An amount of 200 μl was replaced using the pipette into the 96-well microplates in the same way. Readings were taken using the microplate reader at a 595-nm wavelength. It was noticed that the third well readings of the Durafill, Rok and Venus (corresponding to the organization numbers 7, 13, and 14 in the microtiter plate, respectively) were not consistent with the readings of the other wells for the same material. So, another 200 μl of the third well of each material was taken after mixing the contents and added to another 96-well microplate tube, and readings were retaken for confirmation.

One-way analysis of variance (ANOVA) statistical comparison was used for both the 50-μl and 100-μl added composite saline extract groups with a significance level of 0.05 for the statistical analysis to compare the MTT precipitate absorbance values of the resin composite's extracts to the controls. Data were transformed because Levene's test for equality of variance values was not fulfilled. Thus, log transformation of variables (log 10) was done, and new variables were computed.

## Results and discussion

Infrared spectra of the resin composite samples and their saline extracts were subtracted from their own baselines. For a more accurate evaluation of the intensities of the peaks and fractions of different functional groups, all of the spectra of the saline-soaked samples were subtracted from their own baselines. All of the spectra are baseline corrected by selecting the baseline correction (automatic correction) option. Average readings of the MTT viability testing are presented in Table 1.

**Table 1:** **Average readings of the MTT viability testing**

	50-μl added saline extracts		100-μl added saline extracts	
	Average absorption	Standard deviation ±	Average absorption	Standard deviation ±
	***N***= 3		***N***= 3	
Control	0.145	0.010	0.734	0.100
Prisma AP.H	0.165	0.010	0.309	0.070
4 Seasons	0.146	0.001	0.293	0.070
Tetric Evo Ceram	0.228	0.010	0.241	0.020
Filtek Supreme	0.236	0.010	0.394	0.300
SureFil	0.180	0.010	0.655	0.100
Quixx	0.215	0.010	0.375	0.020
Durafill	0.226	0.010	0.771	0.500
Heliomolar	0.174	0.030	0.283	0.100
Esthet.X	0.170	0.010	0.370	0.100
Grandio	0.158	0.020	0.580	0.050
ICE	0.229	0.030	0.500	0.020
3D-Direct	0.204	0.010	0.766	0.100
Rok	0.198	0.010	0.874	0.200
Venus	0.231	0.030	0.500	0.300

**Figure 4:**
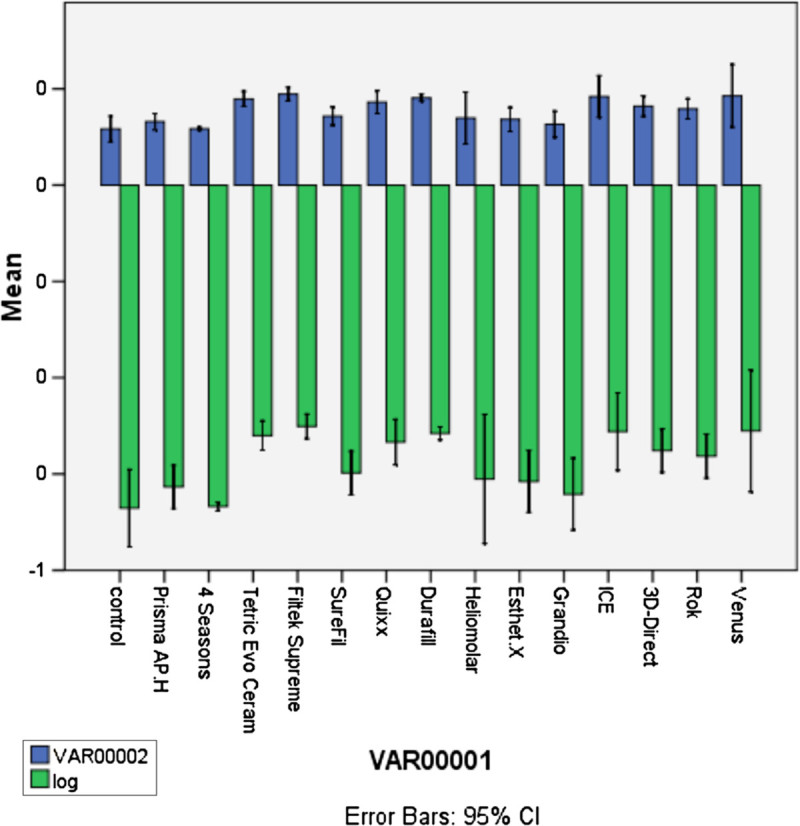
**Statistical analysis of the 50-μl composite saline extract added to the 450-μl cell culture.**

Statistical analysis of the 50-μl composite saline extract added to the 450-μl cell culture is presented in Figure 4. One-way ANOVA of MTT precipitate absorbance readings was calculated. The analysis was significant, F(14,30) = 14.64, *p* < 0.05. The MTT precipitate value was found to be more with Tetric Evo Ceram (mean difference (*M*) = -0.65, standard deviation (SD) = 0.02), Filtek Supreme (*M* = -0.62, SD = 0.01), Quixx (*M* = -0.67, SD = 0.02), Durafill (*M* = -0.64, SD = 0.01), ICE (*M* = -0.64, SD = 0.04), 3D-Direct (*M* = -0.69, SD = 0.02), Rok (*M* = -0.70, SD = 0.02), and Venus (*M* = -0.4, SD = 0.06) as compared to the control (*M* = -0.84, SD = 0.04). It was noticed that the mean difference values were negative, which means that the above-mentioned resin composite's extracts have higher MTT precipitate absorbance than the control and thus higher metabolic activity (usually taken to equal viability) values.

**Figure 5:**
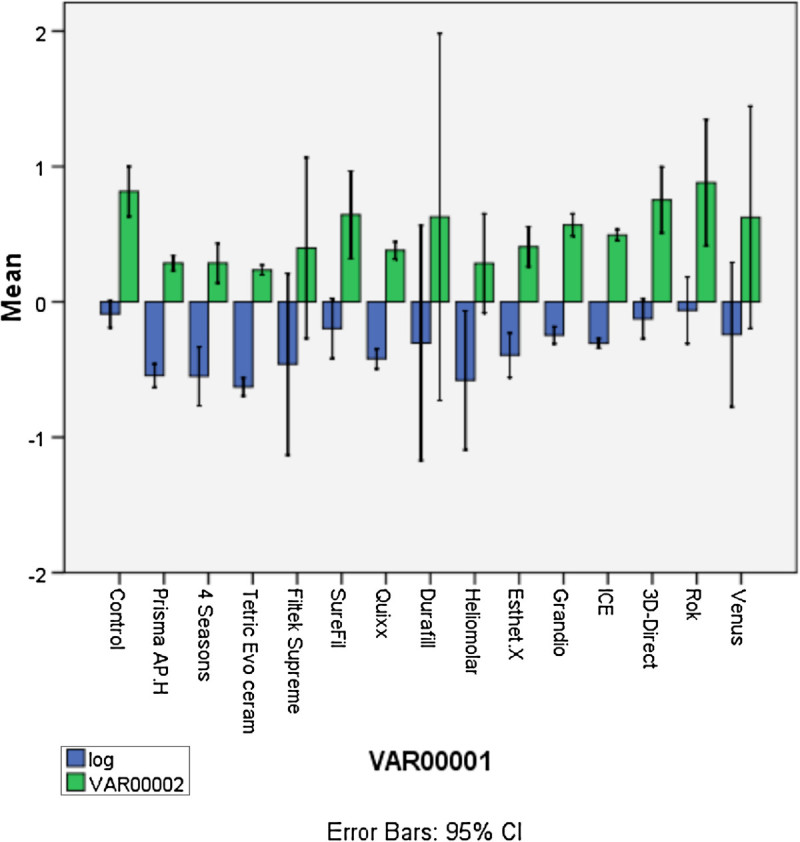
**Statistical analysis of the 100-μl composite saline extract added to the 400-μl cell culture.**

Statistical analysis of the 100-μl composite saline extract added to the 400-μl cell culture is presented in Figure 5. One-way ANOVA of MTT precipitate absorbance readings was calculated. The analysis was significant, F(14,30) = 4.75, *p* < 0.05. The MTT precipitate value was found to be less with Prisma AP.H (*M* = -0.55, SD = 0.03), 4 Seasons (*M* = -0.55, SD = 0.09), Tetric Evo Ceram (*M* = -0.63, SD = 0.03), and Heliomolar (*M* = -0.58, SD = 0.21) as compared to the control (*M* = -0.09, SD = 0.04). It was noticed that the mean difference values were positive, which means that the above-mentioned resin composite's extracts have lower MTT precipitate absorbance than the control and thus statistically higher cytotoxic effects.

### Infrared spectroscopic analysis for the transmittance spectra

When quickly viewing the spectra of the resin composites as is, the spectra of all the resin composite brands look almost identical. They have the same general band positions, and the differences between them seem minute or even null. More careful analysis is required in using IR spectroscopy as a tool for differentiating as-prepared resin composite brands.

After analyzing the spectra of the saline-soaked samples, there was a significant change in the intensity of the peaks of all of the resin composite brands. This intensity differs among brands, with the most reduction in intensity shown in Esthet.X and Durafill and minimal reduction shown in SureFil and 3D-Direct. The difference in peak intensity reduction among resin composite brands could provide a valuable differentiation tool for forensic purposes in that the saline-soaked samples resemble the resin composite restorations more after placement in the patients' mouths than the as-prepared resins. For that reason, better quantitative analysis of the spectra can be achieved by plotting the spectra in absorbance (Smith 1998).

When analyzing the spectra of the saline extracts, it was noticed that resin composite brands have different leaching abilities as some resin composite brands' saline extracts had more intense peaks than the others. The most intense peaks were shown in Durafill, Esthet.X, and Venus saline extracts, and minimal or even no peaks were shown in Grandio and Heliomolar saline extracts. It was also noticed that after distilled water leaching and distilled water rinsing, all composite saline extract spectra had lost the peaks eventually except in 3D-Direct and ICE saline extracts. For better quantitative analysis of the saline-extracted materials, the spectra of the saline extracts were also plotted in absorbance. As found in previously published analyses of the inorganic elemental compositions of composite resins, there are small but useful discriminating features in their IR spectra characterizing their covalently bound resin and filler components.

### IR spectroscopic analysis for the absorbance spectra of the saline-soaked samples

After saline soaking, the IR spectra of the samples showed that all peak positions remained the same, but there was decrease in the intensity of all peaks, which was different among the resin composite brands. The surface characteristics and composition of the saline-soaked samples are believed to be of more interest to study as it resembles the surface of the resin composite restorations after placement in the patients' mouths.

After comparing the shapes of the bands for the absorbance spectra of the saline-soaked samples, it was noticed that 3D-Direct, Rok, ICE, 4 Seasons, Tetric Evo Ceram, Venus, and Grandio have similar band shape in the region between 1,200 and 600 cm^-1^ (silica stretch region). Quixx has a unique band shape in the region of 1,200 to 600 cm^-1^. Esthet.X and Prisma AP.H have similar bands shape in the region between 1,200 and 600 cm^-1^. Filtek Supreme, Heliomolar, and Durafill have similar band shape in the region between 1,200 and 600 cm^-1^, and they have a unique intense peak at 800 cm^-1^, yet to be correlated with specific filler components. SureFil has a similar band shape as Filtek Supreme, Heliomolar, and Durafill, but the band at 800 cm^-1^ is less accentuated. From the above qualitative comparison of the bands' shapes among the 14 dental resin composites, it is found that it is possible to categorize resin composite brands according to the shapes of their infrared spectra, at a qualitative ‘pattern recognition’ level. This finding can help and would add to the database to aid future and forensic discrimination among different dental resin composite brands.

For quantitative comparison, the two major bands (ester band at ≈1,700 cm^-1^ and silicate band at 1,200 to 800 cm^-1^) were compared in all the absorbance spectra of the saline-soaked samples. Also, comparison of the fraction of the ester band absorbance to the silica band absorbance was made, and it was found that 3D-Direct has the highest ester band absorbance among all other resin composite brands with an absorbance value of ≈0.4, followed by 4 Seasons and Prisma AP.H with a value of ≈0.2. Heliomolar, Rok, SureFil, Grandio, Quixx, Tetric Evo Ceram, and Venus have an ester band absorbance value of ≈0.1. The other resin composite brands have lower ester band absorbance values. It was also found that the highest silica band absorbance was for 3D-Direct too, with an absorbance value of ≈1.0, followed by SureFil and Heliomolar with a silica band absorbance value of ≈0.5. 4 Seasons showed a silica band absorbance value of ≈0.4. Tetric Evo Ceram and Grandio have a silica band absorbance value of ≈0.3, followed by Durafill, Filtek Supreme, Rok, Prisma AP.H, and Venus with a silica band absorbance value of ≈0.2. Esthet.X, ICE, and Quixx were found to have the lowest silica band absorbance among all resin composite brands. These findings show that quantitative difference in band absorbance among the saline-soaked resin composite samples does exist.

The ester/silicate absorbance ratio value represents the fraction of the major organic band to the major inorganic band absorbance. The ester/silicate absorbance values were found to be highest for the resin composite brand Quixx with a value of ≈0.6, followed by Prisma AP.H, 4 Seasons, ICE, and Rok with a value of ≈0.5 and 3D-Direct with a value of ≈0.4. Tetric Evo Ceram, SureFil, Esthet.X, Grandio, and Venus showed a value of ≈0.3. Filtek Supreme, Durafill, and Heliomolar have the lowest fraction of organic ester/inorganic silicate absorbance.

From the above qualitative and quantitative comparisons of the absorbance spectra of the saline-soaked resin composite samples, it was found that the resin composite brands could be categorized into similar or different groups. This can be used as a valuable tool to differentiate resin composite brands for forensic purposes using IR spectroscopic analysis.

### IR spectra of the pure basic materials

The pure basic materials (urethane dimethacrylate, TEGDMA, bis-GMA, and camphorquinones) are the main materials present in the composition of most of the resin composite brands as supplied by the manufacturers (Air Force Medical Services Public Site, 2012). These mixtures comprise the monomers and photoinitiators. Other materials constituting the composition of the dental resin composite brands are the different fillers. Many studies have been done to study the effects of the monomers in their pure forms on the cellular viability and mutational effects (Schmalz et al. 1999; Janke et al. 2003; Issa et al. 2004; Theilig et al. 2000; Moharamzadeh et al. 2007; Lai et al. 2004).

Upon taking the spectra of different pure basic materials (EDMA, EGDMA, TEGDMA, and bis-A) and photoinitiators (camphorquinones), it was found that the spectra look almost the same as each other. That explains why dental resin composite materials with different combinations of some of these mixtures still look almost the same. It was also noticed that some of these pure material spectra have the same band positions found in the resin composite spectra but with sharper and more intense peaks in the low molecular size pure materials. This could be explained by the fact that dental resin composite surface composition is a polymerized mixture of materials, so the presence of other bands and convolution of the bands are a logical explanation of the wider and convoluted band spectra. Also, the spectra of the pure materials are missing the wide band at 1,200 to 800 cm^-1^, which corresponds to the silica stretch found in the dental resin composites. The silica stretch found in the dental resin composites is due to the presence of the inorganic filler particles.

### Absorbance spectra of the saline extracts

When the absorbance spectra of the composite saline extracts were evaluated, it was found that different resin composites have different leaching abilities according to the different absorbance bands present in some of the extracts and not present in others. The resin composite brands with intense saline extract absorbance bands are 4 Seasons, Durafill, Prisma AP.H, Quixx, SureFil, and Venus. Although these composites showed the most intense bands, this finding cannot be correlated to the MTT viability findings presented in Table 1 because Durafill, SureFil, and Venus were shown to have minimal or no cytotoxicity to HGF cells when 100 μl of their extracts was added, even though they are having what appeared to be the most leaching materials.

For that reason, quantitative analysis of the absorbance of the major bands was carried out and confirmed the absence of correlation between the band absorbance and viability findings. The three major peaks found in the saline extract spectra are at ≈1,718 cm^-1^, two peaks with 1:1 ratio at 1,318 and 1,294 cm^-1^, and at 1,168 cm^-1^ corresponding to ester bond, aromatic amines, and carboxylic acids/esters, respectively (Smith 1998).

The band at 1,718 cm^-1^ is most intense in Venus followed by 4 Seasons, Durafill, Filtek Supreme, ICE, Prisma AP.H, Quixx, and SureFil. The two peaks at 1,318 and 1,294 cm^-1^ are most intense in Venus followed by 4 Seasons, Durafill, Prisma AP.H, and SureFil. The band at 1,168 cm^-1^ is present in 4 Seasons, Durafill, Prisma AP.H, Quixx, SureFil, and Venus. From the previous findings, it was shown that Venus, 4 Seasons, Durafill, Prisma AP.H, and SureFil have the most leaching abilities. Thus, different composite resin brands have different leaching abilities. Also, it was determined that saline-extractable components of these same resins can have differential effects on the viabilities of HGFCs and that such effects are likely to be concentration dependent.

### SEM/EDS of the saline extract

**Figure 6:**
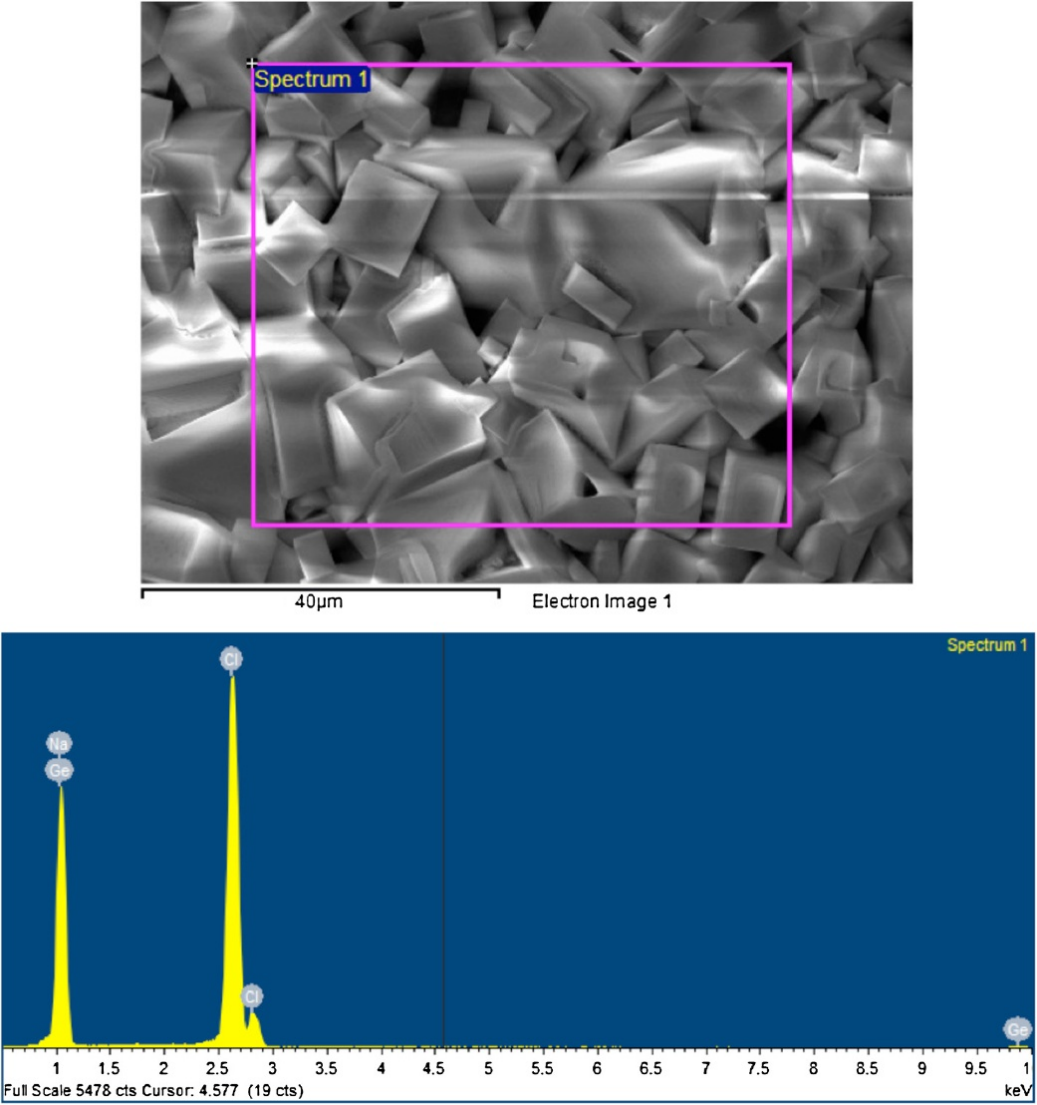
**The composite saline extract deposits.** The upper panel is the SEM image of the composite saline extract deposits. The lower panel is the EDS finding of the elemental analysis of the deposit.

**Figure 7:**
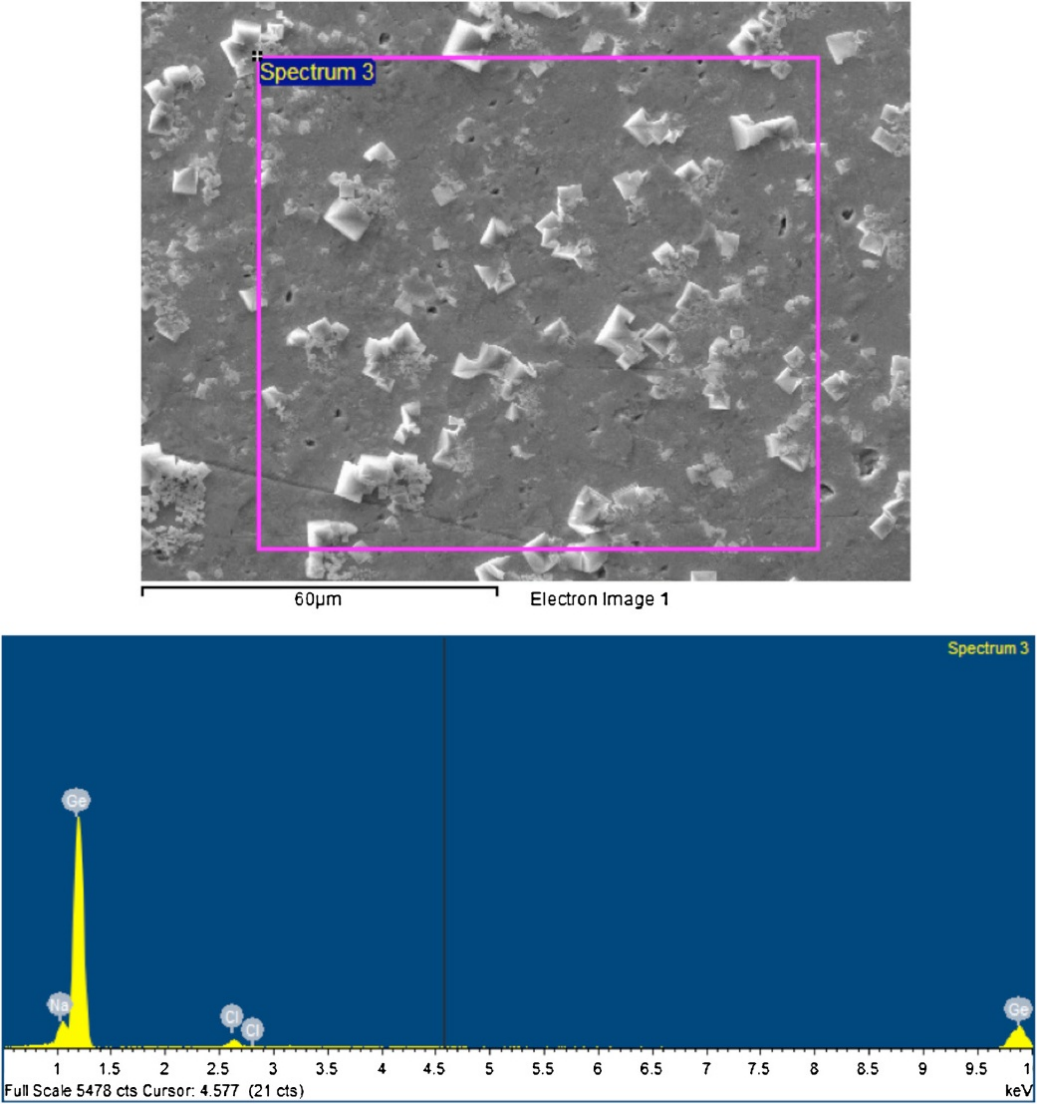
**Germanium prism surface with some composite saline extract deposits.** The upper panel is the SEM image of the germanium prism surface with some composite saline extract deposits. The lower photo is the EDS finding of the elemental analysis of this surface.

Previous studies were done about leaching of fillers from dental resin composites in distilled water (Soderholm 1983, 1990, 1981). These studies were done using distilled water as the incubation media and concluded that filler particles do leach. None of the resin composites brands used in these studies were the same brands as those used in our study. To investigate whether the filler particles might leach in the saline extract, unfiltered resin composite saline extract dried on germanium prism was analyzed using SEM/EDS and showed only Na, Cl, and Ge (Figures 6 and 7). This might indicate either that no inorganic fillers leach from the resin composite or that the amount of fillers leached is very minute or was skipped during EDS analysis. Also, the leaching of the fillers might be time dependent as the previous studies were done in a 30-day to 6-month period while only 2 weeks of incubation period was used in our study.

### MTT viability test

MTT viability assay was done to find out if the composite saline extracts have different cytotoxic effects on HGF cells as it was found that they possess different leaching abilities. One-way ANOVA was used to compare the viability values of the composite saline extracts to the control in each group (the 50-μl added and the 100-μl added composite saline extracts). It is not possible to compare the values between the two groups because each experiment was done in separate cultures on different days. Even though all factors were standardized, different cell lines would have different proliferation rates, and their behavior is not predictable. This explains the difference in the absorbance readings of the controls of both groups. However, comparison between the two groups can be carried out according to how they differ from their own control.

When analyzing the results of the 50-μl added composite saline extract group, it was noticed that there was statistically significant different values between the control and Tetric Evo Ceram, Filtek Supreme, Quixx, Durafill, ICE, 3D-Direct, Rok, and Venus. It was also noticed that these materials had significantly higher MTT precipitate absorbance values than the control. This can be explained either due to the low sensitivity of the MTT test or because of the fact that the MTT test is actually a measure of mitochondrial activity rather than true cell viability, and the addition of a low amount of cytotoxic materials not sufficient to kill the cells will cause the cells to metabolize these toxins and thus increase the mitochondrial activity. Another possible explanation could be derived from the science of homeopathy. Homeopathy is based on the idea that small doses of a substance that would cause symptoms when administered in large doses will actually activate the defense mechanism against this substance (American Cancer Society, 2012). This can possibly explain why in our studies there was an increase in the cell viability results when low doses of resin composite extracts were administered to HGF cell cultures.

When analyzing the results of the 100-μl composite saline extract, it was found that there were significantly different values of the MTT precipitate readings but in contrast to the 50-μl added resin composite saline extract group. These results indicate lower MTT precipitate absorbance values, which indicate lower viability results. These significantly different values were shown by Prisma AP.H, 4 Seasons, Tetric Evo Ceram, and Heliomolar. When compared to the control from the above findings, it was noticed that different resin composite brands do leach materials that possess different cytotoxic effects to HGF cells and that this cytotoxic effect is threshold dependent.

### Limitations of this study

Inorganic filler particles in dental composites can leach ions from compounds of silicon, barium, strontium, and sodium (Soderholm 1981, 1983; Soderholm et al. 1984), but it is also likely that those detected elements actually could be present in compounds such as silicates and carbonates that do have IR-detectable covalent bonds. MAIR-IR spectrometry used in our study detects such functional groups and most other covalent bonds (Smith 1998) but will not detect inorganic leaching ions from filler particles, which might also affect cells in the proximal vicinity of the resin composite restorations in the mouth.

Saline at body temperature was used in correspondence to previous laboratory work on other restorative materials (Intermediate Restorative Material (IRM), Geriostore, and Ketac Fil) to study the leachable materials from these dental restoratives (Al-Sabek et al. 2005). Also, saline is harmless to cells and does not give any readings in the MAIR-IR spectrometer (because it contains only ionically bonded salt). This is one of the biggest limitations encountered in our study because the use of saline alone may not absolutely mimic the more complex *in vivo* oral environment in which these resin composite restorations are placed. The oral cavity is subjected to different chemistries frequently during eating of food and drinking of various beverages. Also, food and drinks will subject the oral cavity to major fluctuations in temperatures and degrees of abrasion. All of the changes that occur in the oral cavity can affect the degree and amount of leaching materials from dental resin composite restorations placed in it. Also, the oral environment is subject to the deposition of different amounts of plaque and calculus that consequently may absorb the leaching materials and so affect the duration and frequency of exposure of cells adjacent to the retained debris on these materials (Lee et al. 1995a, 1995b, 1998). Other than foods and drinks, saliva does contain enzymes, and hydrolysis and/or enzyme catalysis can also cause chemical degradation of dental composites (Koin et al. 2008). These factors must be considered although it is difficult to standardize all of these factors as they are not controlled and differ from person to person due to natural differences among people and lifestyles.

Many biological reactions *in vivo* are not immediately cytotoxic and are extended well beyond 24 h. Cytotoxicity assays measure mainly finite effects on cells during the first 12 to 24 h after exposure to toxic substances and are the major category of tests designed for the initial evaluation of materials. Other important processes that should be taken into consideration are inflammation, immune reactions, and mutagenesis for comprehensive testing of the effect of these materials on cells and to more clearly postulate what will happen in the real human model (Hanks et al. 1996).

Depending on only one testing method for ideal surface analysis, characterization, and comparison is not possible. The use of other techniques and adding the results together are very important. Other techniques could be SEM/EDS (Bush et al. 2008; Ubelaker et al. 2002; Hosoda et al. 1990), contact angle goniometry (Galan et al. 2004), XRF (Bush et al. 2007b, 2008), or quantitative light-induced fluorescence (Pretty et al. 2002).

Cells might come into direct contact with these resin restorative materials (e.g., periodontal ligament (PDL) fibroblasts in root-end filling materials, dental pulp fibroblasts in direct pulp capping, gingival fibroblasts in class IV subgingival restorations, and buccal and labial mucosa in bonding resins of orthodontic brackets). Better knowledge of surface characteristics will be crucial for better understanding of how cells in direct contact with these restorations will react. Huang et al. (2002) stated that resinous perforation repair materials inhibit the growth, attachment, and proliferation of human gingival fibroblasts. The study of Al-Sabek et al. (2005) showed preferential attachment of HGFs to the resin ionomer Geriostore when compared with IRM and Ketac Fil but did not explain the reasons for the results. Another study did direct-contact cytotoxicity testing of resin-based restoration materials on HGFs and resulted in finding a time-dependent reduction of their growth with irritation and defective morphology of the fibroblasts in the vicinity of the resin-based materials (Willershausen et al. 1999). Sailynoja et al. (2004) used both direct-contact and extract methods for cytotoxicity testing of UTMA-based hybrid resin and concluded that with increasing incubation temperature to 72°C, cytotoxic effects of the extracts were shown whereas the lower-temperature extracts did not, and that the direct-contact test did not show cytotoxicity. Tuncel et al. (2006) used an agar diffusion method, and cytotoxicity rankings were determined using lysis index scores for cytotoxicity evaluation of three different composites. The study found that the cytotoxicity of the composites increased when fiber reinforced. No chemical analysis of the cytotoxic elements was provided, however.

Another limitation to any *in vitro* model of ‘biocompatibility’ is the time allotted for incubation of the samples in saline or other media. Although 2 weeks was enough to produce a sufficient amount of leaching materials to be analyzed by IR spectroscopy, it is likely that all commercially available resin-based dental materials will continue to release components that may cause detrimental effects or alter cellular function *in vitro* even after 2 weeks of aging in artificial saliva. Wataha et al. (1999) call attention to the effect of chronic exposure of the cells *in vivo* to these materials with continuous wash out when swallowing versus the one-time subjection of the cells *in vitro* to 2-week accumulated leaching materials.

HGFs were chosen for this study because they are cells in proximal vicinity to dental restorations. PDL fibroblasts would also be affected, could simulate the periapical tissues even better, and are known to be similar to gingival fibroblasts, except that they have a higher production rate of collagen. Also, gingival fibroblasts were chosen due to their easy availability and culturing characteristics (Huang et al. 2002; Hou and Yaeger 1993).

## Conclusions

Different resin composite brands have interestingly different surface characteristics after incubation in saline, which were not as readily found in the materials as is. This finding made it possible to categorize the saline-soaked resin composite brands according to their absorbance spectra shapes and values. This might be beneficial addition to the database for forensic discrimination and characterization of different resin composite brands according to a new method, which is IR spectroscopic analysis. It will also fill the gap of studying the organic portion that was not covered by the previous studies, which were concentrating on the inorganic portion.

The fact that the saline-soaked samples were found to have different spectra from the as-is samples and from each other raised the value of studying these resin composite surfaces after incubation in fluids that will more closely simulate the oral environment with fluctuating temperatures and acidity. These fluctuations can be due to different eating and drinking habits. Also, the restorations, when placed in the oral cavity, are subjected to frictional forces and deposition of plaque and calculus that will act on them and change their surface chemistry. All of the above factors should be considered in future studies for better understanding of the surface characteristics of these resin composite brands inside the patients' mouths.

For the biotoxicity aspect, the IR spectra for the saline-soaked samples showed changes in surface characteristics of resin composites. This is of great importance to study as this surface is in contact with oral mucosal cells and was not attended by most of the previous studies. Also, the IR spectroscopy of the saline extract showed that different resin composite brands would have different leaching abilities although these findings are not well correlated to the viability findings. This makes it crucial for future studies to find the correlation between the leached materials and cytotoxicity findings, which was found to be threshold dependent. For the biological effects of these resin composites on the HGF cells, the oral environmental factors mentioned earlier should be considered, and the application of direct viability test and also more than one surface characterization technique are essential for better understanding of the biological effects of resin composite brands to cells in proximal vicinity to them in the oral cavity.

More sensitive and precise viability testing methods in combination with more clinically relevant situations should be the target of future studies. This study focused on differentiating different resin composite brands, which was not the case in previous studies. Previous studies focused on the difference between composites and other restorative dental materials and did not address the wide variety of dental resin composite brands, which were proven by this study to have different surface characteristics and biological behaviors.

Also, the conclusion from these studies is that IR spectrometry (particularly using the very surface-sensitive MAIR technique) can provide valuable reference characteristics for later forensic identification of the distinct resin composites present in unknown trauma victims. MAIR-IR can also identify miniscule amounts of saline-extractable components from resin components that can have differential consequences for the viabilities of neighboring gingival fibroblasts. It should be a new requirement for such analyses that IR spectroscopic identification be attempted given these early successes.

## Authors’ original submitted files for images

Below are the links to the authors’ original submitted files for images. Authors’ original file for figure 1 Authors’ original file for figure 2 Authors’ original file for figure 3 Authors’ original file for figure 4 Authors’ original file for figure 5 Authors’ original file for figure 6 Authors’ original file for figure 7 Authors’ original file for figure 8 Authors’ original file for figure 9 Authors’ original file for figure 10 Authors’ original file for figure 11 Authors’ original file for figure 12 Authors’ original file for figure 13 Authors’ original file for figure 14

## Abbreviations

ANOVA: analysis of variance test statistics
BAD or bis-GMA: bisphenol-A dimethacrylate
BPA: bisphenol-A
EDMA: ethylene dimethacrylate
EDS: energy-dispersive spectroscopy
HGF: human gingival fibroblast
IR: infrared
MAIR-IR: multiple attenuated internal reflection-infrared spectroscopy
MTT: methylthiazol tetrazolium, yellow dye utilized by cells as an *in vitro* test of viability and proliferation
PDL: periodontal ligament
SEM: scanning electron microscopy
TEGDMA: triethylene glycol dimethacrylate
XRF: X-ray fluorescence.
