# Supplementary figures and images for: Infrared spectroscopic analysis of restorative composite materials' surfaces and their saline extracts
Source: Prog Biomater. 2013 Mar 18;2:9. doi: 10.1186/2194-0517-2-9 (PMC5151121; doi:10.1186/2194-0517-2-9)

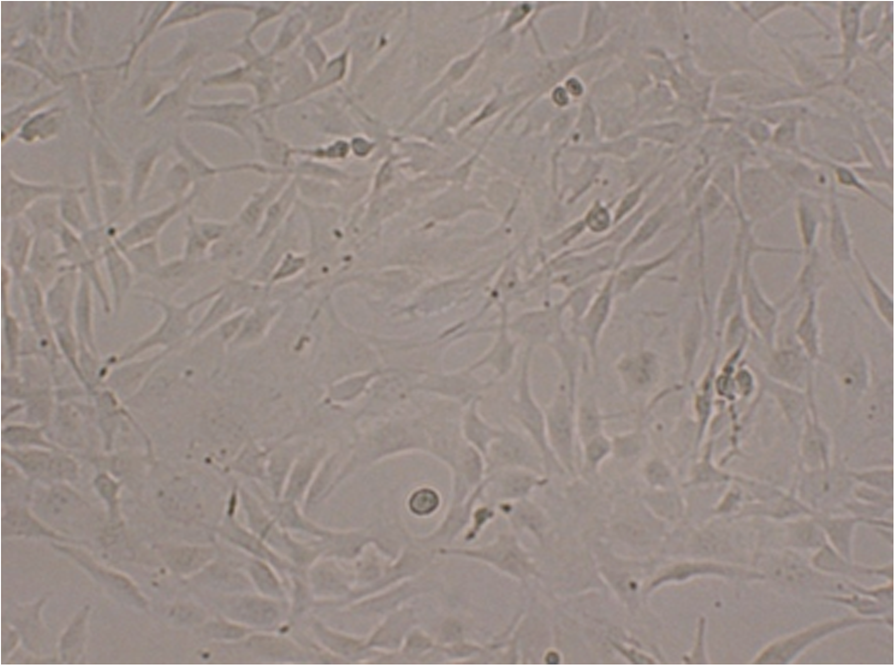

Supplement: Supplementary file 1 — Authors’ original file for figure 1 [file 40204_2012_13_MOESM1_ESM.tiff]

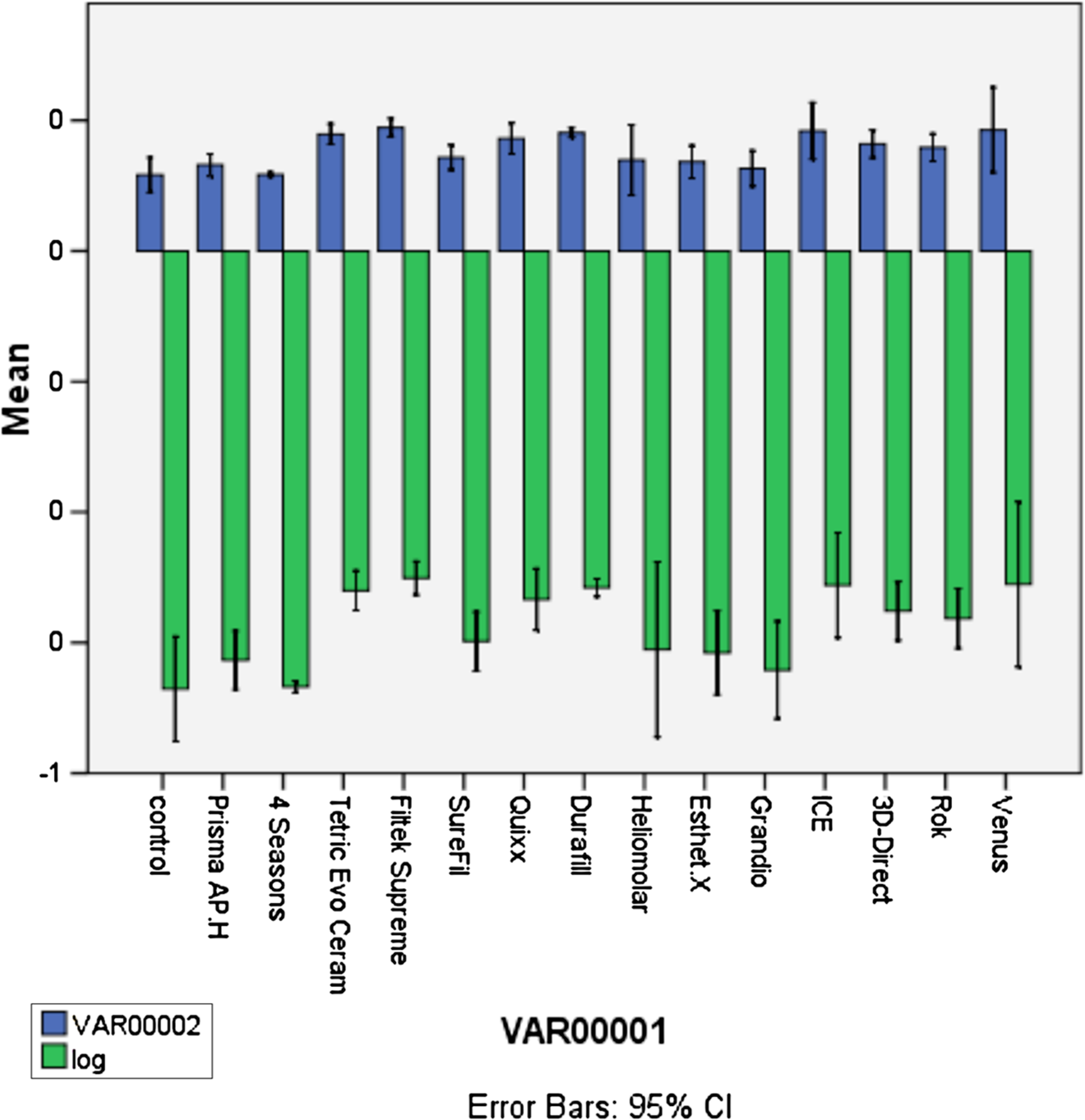

Supplement: Supplementary file 4 — Authors’ original file for figure 4 [file 40204_2012_13_MOESM4_ESM.tiff]

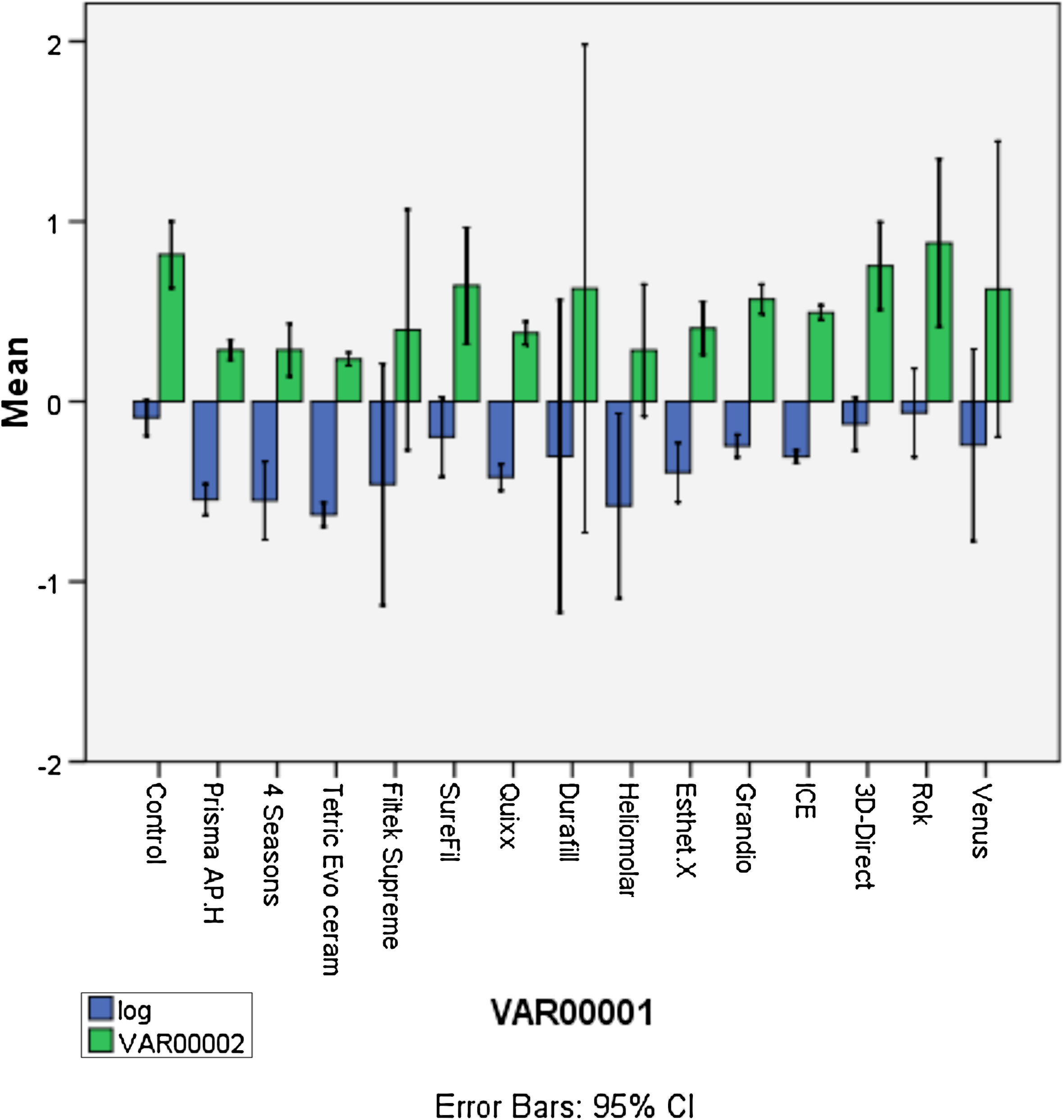

Supplement: Supplementary file 5 — Authors’ original file for figure 5 [file 40204_2012_13_MOESM5_ESM.tiff]

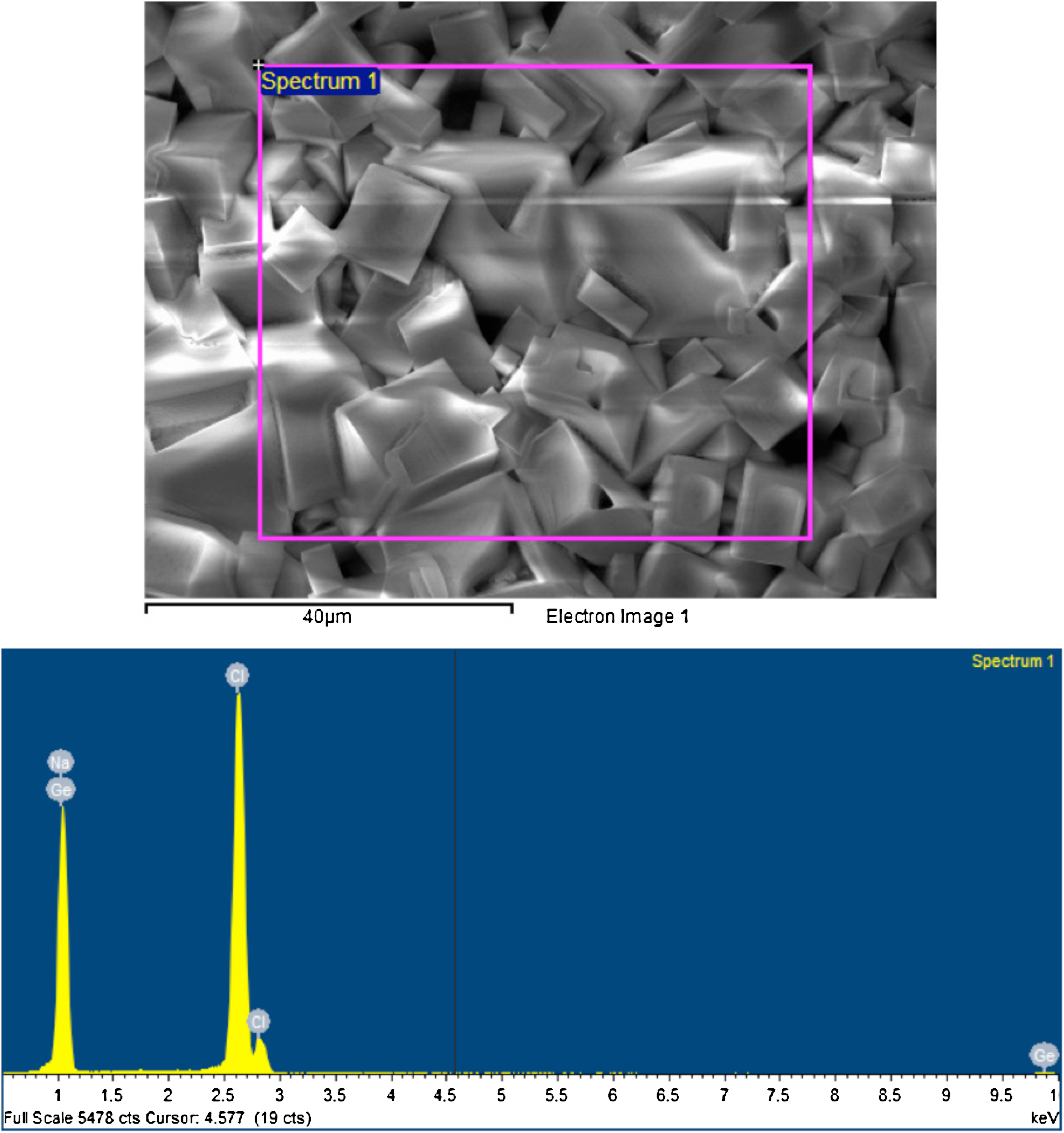

Supplement: Supplementary file 6 — Authors’ original file for figure 6 [file 40204_2012_13_MOESM6_ESM.tiff]

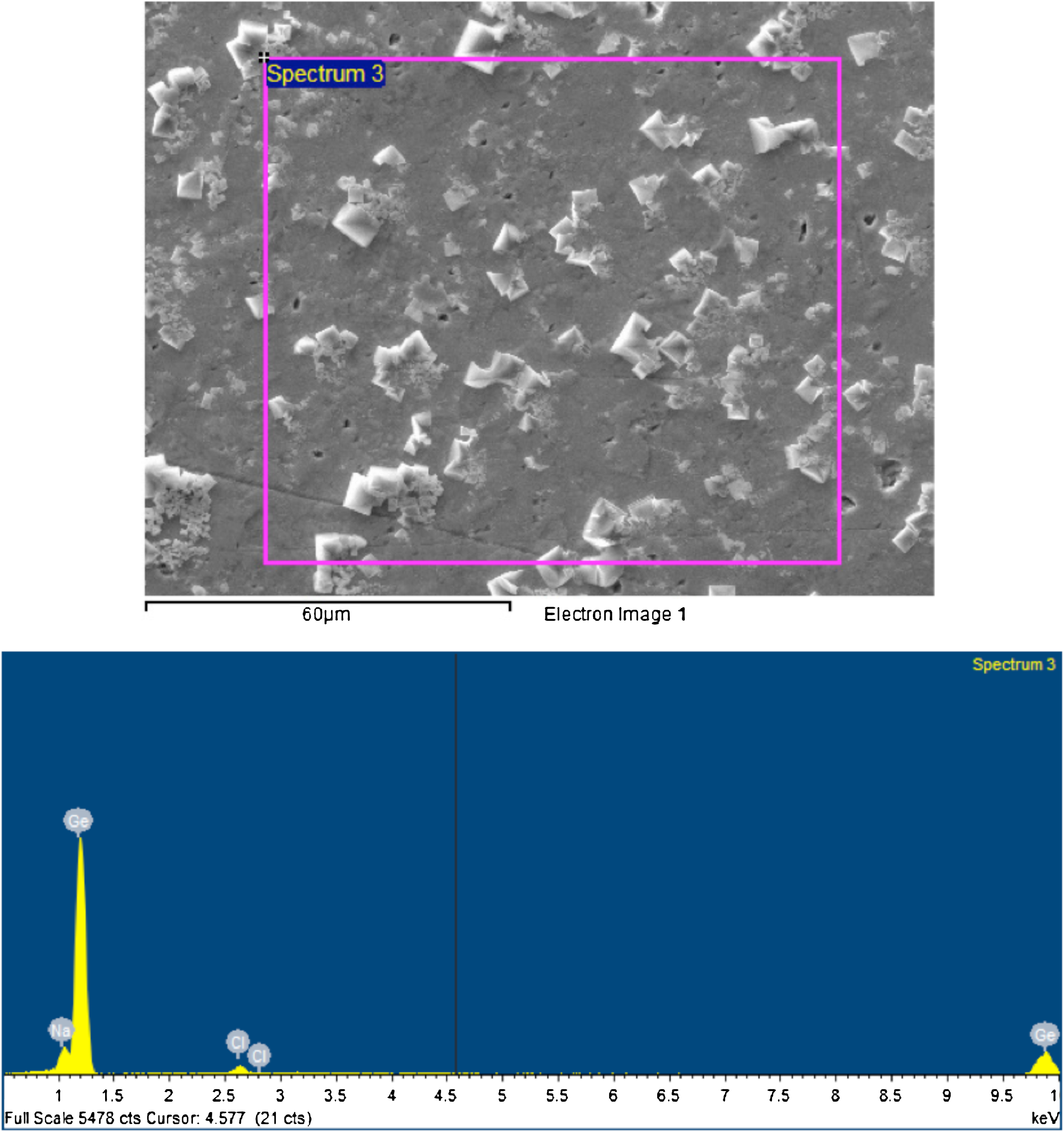

Supplement: Supplementary file 7 — Authors’ original file for figure 7 [file 40204_2012_13_MOESM7_ESM.tiff]

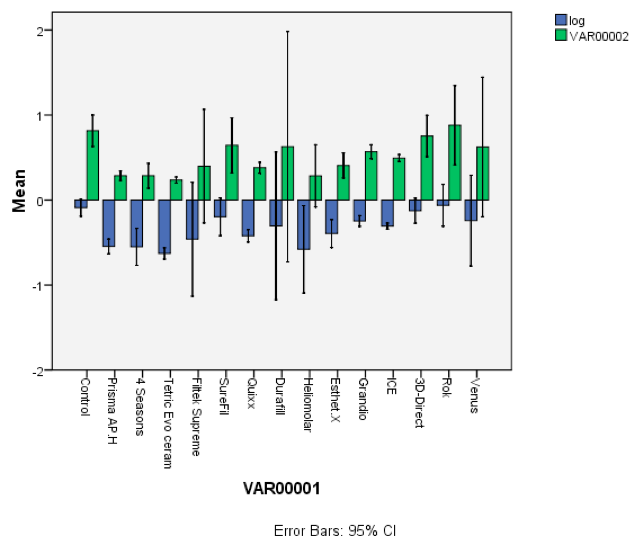

Supplement: Supplementary file 8 — Authors’ original file for figure 8 [file 40204_2012_13_MOESM8_ESM.png]
