# Supplementary material for: Infrared spectroscopic analysis of restorative composite materials' surfaces and their saline extracts
Source: Prog Biomater. 2013 Mar 18;2:9. doi: 10.1186/2194-0517-2-9 (PMC5151121; doi:10.1186/2194-0517-2-9)
Supplement: Supplementary file 2 — Authors’ original file for figure 2 [file 40204_2012_13_MOESM2_ESM.pdf]

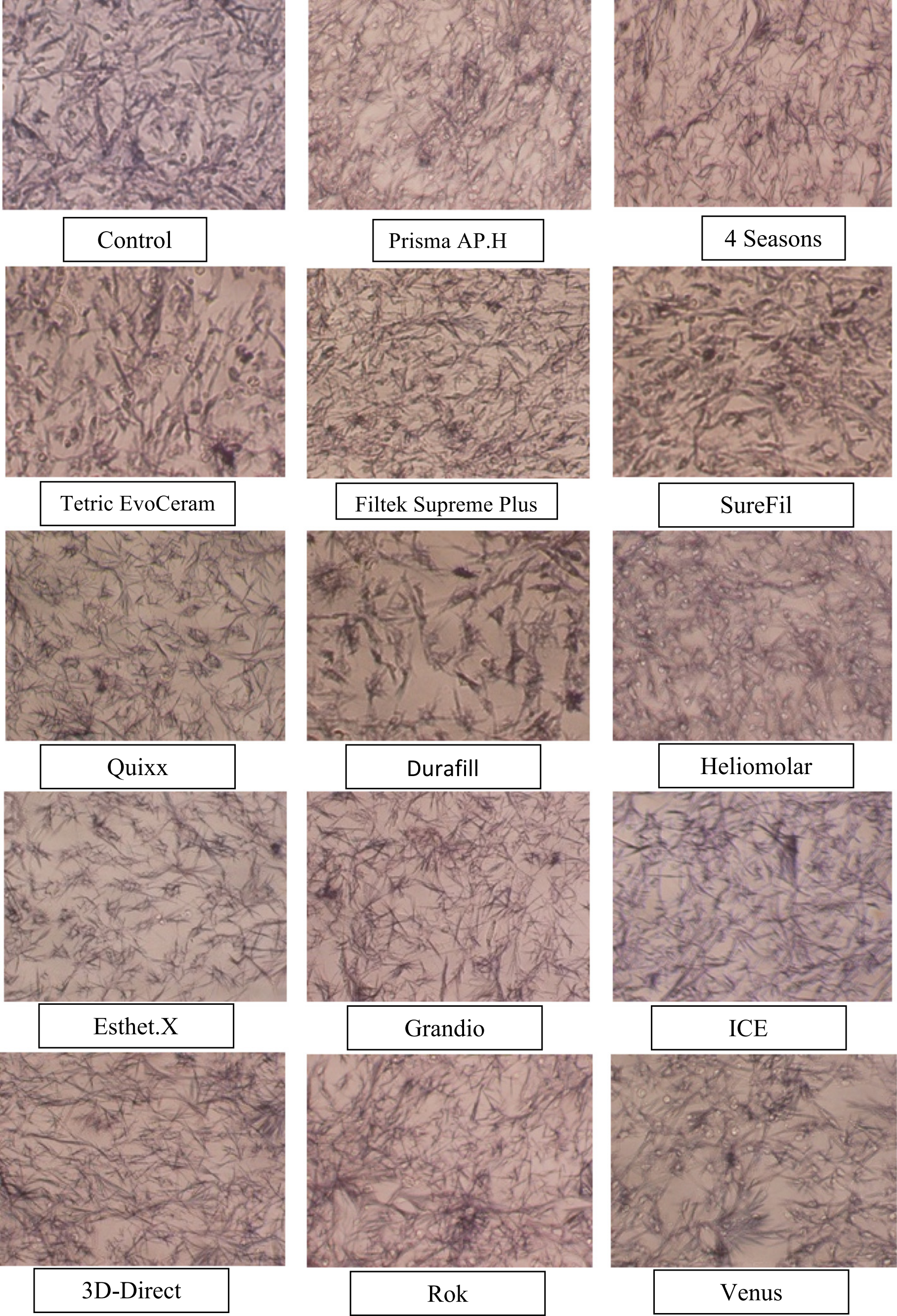
Control
Prisma AP.H
4 Seasons
Tetric EvoCeram
Filtek Supreme Plus
SureFil
Quixx
Durafill
Heliomolar
Esthet.X
Grandio
ICE
3D-Direct
Rok
Venus
