# Supplementary material for: Infrared spectroscopic analysis of restorative composite materials' surfaces and their saline extracts
Source: Prog Biomater. 2013 Mar 18;2:9. doi: 10.1186/2194-0517-2-9 (PMC5151121; doi:10.1186/2194-0517-2-9)
Supplement: Supplementary file 3 — Authors’ original file for figure 3 [file 40204_2012_13_MOESM3_ESM.pdf]

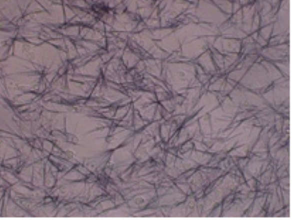

Control

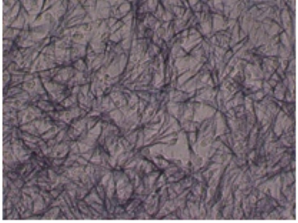

Prisma AP.H

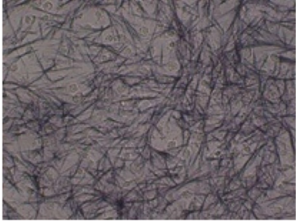

4 Seasons

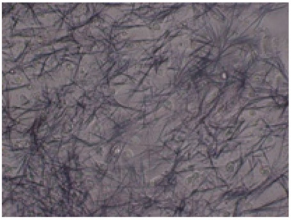

Tetric EvoCeram

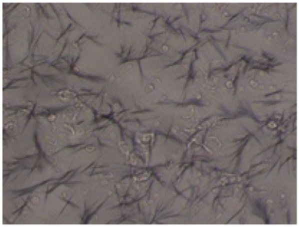

Filtek Supreme Plus

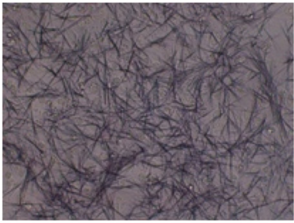

SureFil

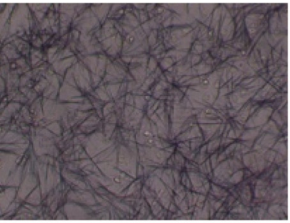

Quixx

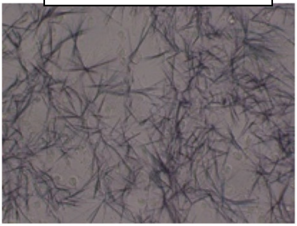

Durafill

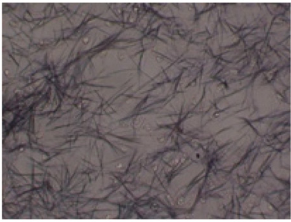

Heliomolar

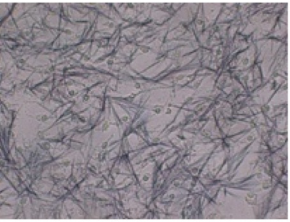

Esthet.X

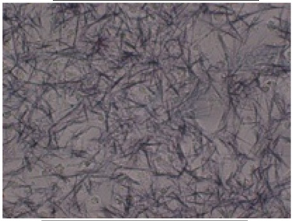

Grandio

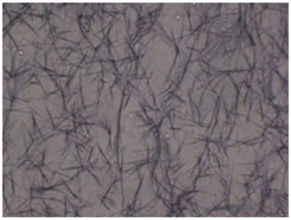

ICE

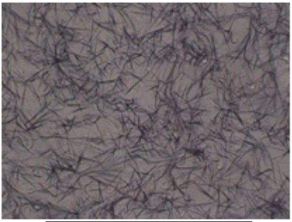

3D-Direct

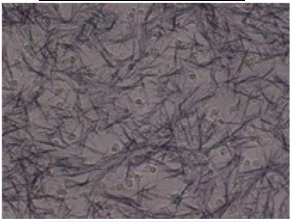

Rok

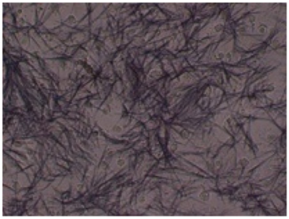

Venus
